# Determination of vitamin D in tears of healthy individuals by the electrochemiluminescence method

**DOI:** 10.1002/jcla.22830

**Published:** 2019-01-21

**Authors:** Yu Tsun Lai, Renato Galão Cerquinho, Matheus Moreira Perez, Beatriz da Costa Aguiar Alves, Edimar Cristiano Pereira, Ligia Ajaime Azzalis, Virginia Berlanga Campos Junqueira, Lismeia Raimundo Soares, Fernando Luiz Affonso Fonseca

**Affiliations:** ^1^ Laboratório de Análises Clínicas da Faculdade de Medicina do ABC Santo André Brazil; ^2^ Departamento de Oftalmologia da Faculdade de Medicina do ABC Santo André Brazil; ^3^ Departamento de Ciências Farmacêuticas da Universidade Federal de São Paulo (UNIFESP) Diadema Brazil; ^4^ Universidade Federal do Rio de Janeiro (UFRJ) Macaé Brazil

**Keywords:** electrochemiluminescence, peripheral blood, reference values, tears, vitamin D

## Abstract

**Background:**

Vitamin D is a fat‐soluble steroid hormone which can be converted into various forms and is of extreme physiological importance to our body. However, its functions and local metabolic pathways in some organs, such as the eye, have not yet been well studied. We aimed to verify the correlation between vitamin D levels in blood and tear fluid and the possibility of using tear fluid as a biological material for monitoring eye disorders in the future.

**Methods:**

The electrochemiluminescence method was used to examine blood and tear samples collected with Schirmer test strips from 21 individuals without ocular disease.

**Results:**

At the 95% confidence interval, mean tear fluid vitamin D = 37.8 ± 3.6 ng/mL, which is higher than the serum level, with a mean of 30.3 ± 7.7 ng/mL; Lin's concordance correlation coefficient = −0.018 (−0.174; 0.139), Pearson's coefficient = −0.070, and the Bland‐Altman coefficient = −11.12 (−30.40; 8.16). Results were obtained using the program Stata version 11.0.

**Conclusion:**

It is possible to determine vitamin D levels in tear fluid using the electrochemiluminescence method, and as the results do not correlate with blood, there is possibility of using tear fluid as a biological matrix for detection of vitamin D, which may increase the possibilities of new studies in eye disorders.

## 1 INTRODUCTION

Vitamin D is a fat‐soluble steroid hormone of extreme physiological importance to our body, such as modulation of calcium and phosphate homeostasis,^1^ influence on the immune system by the inhibition of proinflammatory cytokines and induction of phenotypic regulatory T cells.^2^

There are two basic forms of vitamin D: cholecalciferol (vitamin D3) and ergocalciferol (vitamin D2). Vitamin D3 can be absorbed through the diet or is synthesized in the skin: exposure to solar rays—more specifically to the ultraviolet ray B—activates the synthesis of the precursor 7‐dihydrocholesterol.^3^ This vitamin is activated by two hydroxylation steps; in the first step, vitamins D3 and D2 obtained through the diet or sun exposure are transported to the liver, where they will be transformed into calcidiol (25‐hydroxyvitamin D), the form of vitamin D storage in the body by the action of cytochrome P450 and 25‐hydroxylase. Thus, the synthesis of the majority of circulating 25‐hydroxyvitamin D3 (25[OH]D3) occurs. The second step occurs in the kidney, where 25[OH]D3 is catalyzed to its active form 1,25‐dihydroxyvitamin D3 (1,25[OH]2D3).^3^

When the body senses the need to act on blood and bone calcium levels, part of this 25‐hydroxyvitamin D is transported to the kidneys, where it will undergo the last metabolization process, becoming calcitriol (1,25‐ hydroxyvitamin D), the active form of vitamin D.

The presence of vitamin D metabolites in tears has also been shown, which possibly serves for the maintenance of the ocular cornea; however, the origin of vitamin D in tear fluid has not yet been clarified.^4^ There are still few studies that examine the presence of vitamin D synthesis and pathways in the eye, and whether this actually plays any role in autocrine vitamin D synthesis function by ocular cells. Certain studies show the expression and functionality of vitamin D3 in human ocular barriers, indicating that vitamin D3 may be important in ocular barrier function and the maintenance of immunological privilege.^3^

Vitamin D deficiency is commonly described as when the serum 25‐hydroxyvitamin D level is less than 20 ng/mL,^5^ which can lead to several types of health problems such as cardiovascular problems, hypertension, and ophthalmic disorders (eg, dry eye syndrome, a common tear film disease and ocular surface that causes discomfort, visual disturbance, tear film instability and potential damage to the ocular surface).^6^, ^7^

There is evidence of the difference between vitamin D levels in blood and tear fluid in the same organism, and this difference may be related to the principle and sensitivity of the methods used to measure 25‐hydroxyvitamin D.^8^, ^9^ It has been found that the amount of this vitamin is higher in tear fluid than in blood in patients with eye diseases. In addition, some studies show that there are contradictions between the serum value of 25‐hydroxyvitamin D with dry eye disorder^10^, ^11^; therefore the measurement of 25‐hydroxyvitamin D in tears may be more relevant for ocular diseases, especially for ocular surface diseases.^12^

Thus, this study aimed to verify the correlation between levels of vitamin D in blood and tear fluid and the possibility of using tear fluid as a biological material for monitoring eye disorders in the future.

## 2 MATERIALS AND METHODS

### 2.1 Samples and collection procedures

Blood and tear samples were collected from people without ocular disease. Tear samples were collected using OPHTHALMOS test strips used in the Schirmer tear test (STT), a method that evaluates whether the eye produces enough tear fluid to remain lubricated. The strips were placed in the lower eyelid pouches and removed after 5 minutes; afterward, they were placed in 0.5 mL plastic tubes containing 250 μL of saline solution. The tubes were stored at temperatures between 2°C and 8°C for a period of 24 hours (overnight) and analyzed later. Blood samples were collected with a separating gel tube (yellow tube). This study was performed in accordance with the ethical standards established by the Declaration of Helsinki and has been approved by Faculdade de Medicina do ABC Ethical Committee; informed consent was obtained from all individual participants included in the study.

### 2.2 Dosage of vitamin D in tear fluid and blood

Quantitative determination of vitamin D from tear fluid and blood samples of the patients in this study was performed by the electrochemiluminescence technique using the Cobas^®^ e411 device, and its calibration curve was corrected by two calibrators with a low value of 1.32 ng/mL and a high value of 45.50 ng/mL.

### 2.3 Statistical analysis

Data were subjected to statistical analysis by the program Stata version 11.0 (StataCorp LLC, College Station, TX, USA), in which the quantitative variables were described by mean, standard deviation, minimum and maximum values, and their 95% confidence interval. A new variable containing the vitamin D values of the tear fluid obtained from the right and left eye was created. For the tear samples obtained from the subject's eyes (n = 7), the mean values obtained for vitamin D were performed. To evaluate the concordance of vitamin D in blood and tear fluid, Lin's Concordance, Pearson's Correlation, Accuracy and Bland & Altman Coefficient of Agreement were used. For all analyzes, a confidence level of 95% was used.

## 3 RESULTS

In this study, 21 people were recruited; 21 blood samples and 29 tear samples were collected of which 16 samples were from the right eye and 13 from the left eye. Tear fluid was collected from both eyes of 8 people, while samples from the others were collected from only one eye.

It was observed that the mean of vitamin D is higher in tear fluid compared to blood. This fact was also observed when comparing vitamin D value between blood and tears from the left and right eye (Table 1).

**Table 1:** Description of serum and tear fluid vitamin D values (ng/mL)

Variables	Mean (SD)	Minimum‐Maximum	CI 95%
Serum vitamin D (n = 21)	30.3 (7.7)	14.5‐41.3	26.8; 33.8
Tear fluid vitamin D (n = 21)	37.8 (3.6)	29.7‐43.3	36.1; 39.4
Vitamin D in right eye (n = 16)	37.9 (3.4)	32.5‐43.3	36.1; 39.7
Vitamin D in left eye (n = 13)	37.1 (4.5)	27.0‐42.6	34.4; 39.8

CI 95%, confidence interval of 95%; SD, standard deviation.

**Figure 1:**
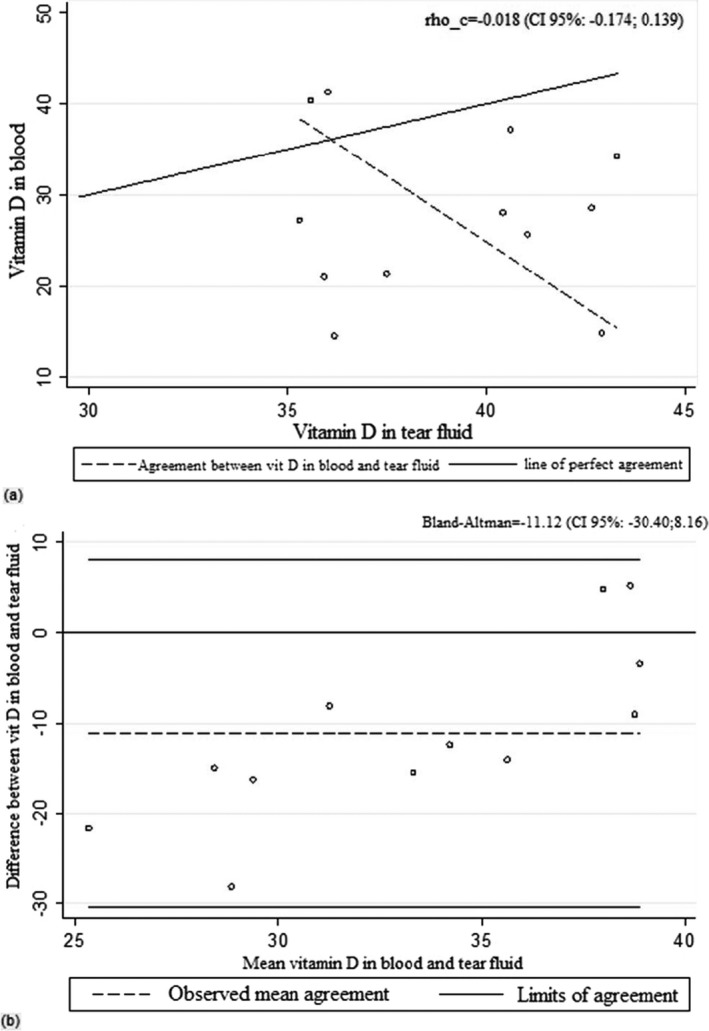
Lin's correlation coefficient (A) and Bland‐Altman (B) for vitamin D in blood and tear fluid

**Figure 2:**
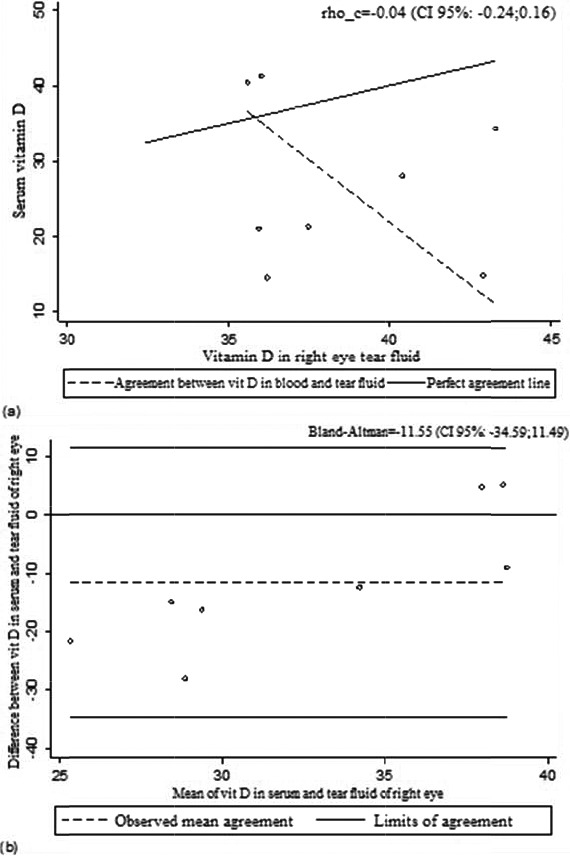
Lin's correlation coefficient (A) and Bland‐Altman (B) for vitamin D in serum and tear fluid of the right eye

**Figure 3:**
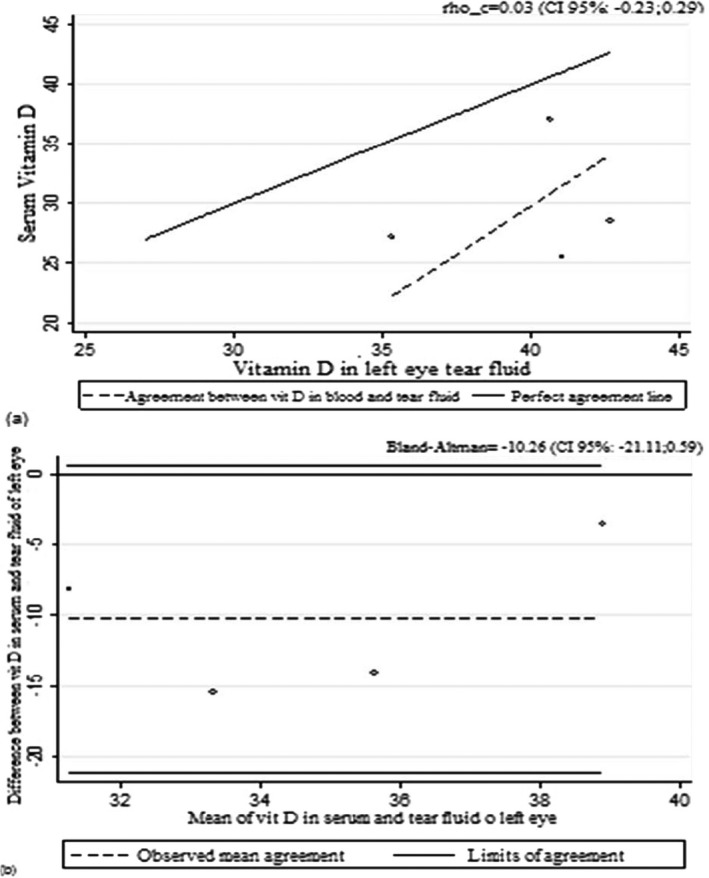
Lin's correlation coefficient (A) and Bland‐Altman (B) for vitamin D in serum and tear fluid of the left eye

When we evaluated the coefficient values of Lin, Pearson, and Bland‐Altman (Tables 2 and 3) and the curves related to the values (Figures 1, 2, 3), it can be deduced that vitamin D values in blood and tears are not correlated in the 95% range.

**Table 2:** Lin's concordance and Bland and Altman's coefficient to measure vitamin D in serum and tear fluid

Variables	Vitamin D			
	rho_c[Link] (CI 95%)	Pearson	Accuracy	Bland‐Altman (CI 95%)
Blood	−0.018 (−0.174; 0.139)	−0.070	0.252	−11.12 (−30.40; 8.16)
Tear fluid				

CI 95%: confidence interval of 95%.

Lin's correlation coefficient.

**Table 3:** Lin's concordance and Bland and Altman's coefficient to measure vitamin D in serum and tear fluid

Variables	Vitamin D			
	rho_c[Link] (CI 95%)	Pearson	Accuracy	Bland‐Altman (CI 95%)
Serum	−0.04 (−0.24; 0.16)	−0.170	0.250	−11.55 (−34.59; 11.49)
Tear fluid in right eye				
Serum	0.03 (−0.23; 0.29)	0.183	0.185	−10.26 (−21.11; 0.59)
Tear fluid in left eye				

CI 95%: confidence interval of 95%.

Lin's correlation coefficient.

## 4 DISCUSSION

Currently, the role of vitamin D in the eye is still not very clear. Some studies examine the physiological functions of vitamin D in a single organ exposed directly to solar rays without being the skin, the eye.^13^

Research on vitamin D testing can be performed by various types of laboratory methods, such as chemiluminescent microparticle immunoassay (CMIA) and ELISA.^12^ Results here described contribute to the observation of the possibility of using the electrochemiluminescence method for the detection of vitamin D levels in both blood and tear fluid. It was observed that the mean level of vitamin D is higher in tear fluid than in the blood in this study, which was also verified in the experiment performed by Sethu et al.^12^ To our knowledge, the cause of this fact and the discrepancy between vitamin D levels of the two biological materials remain unclear.

In terms of tear vitamin D origin, a study demonstrated that the presence of megalin and cubilin proteins in tear and harderian glands in test animals may be involved with ischemic secretion of vitamin D into tear fluid.^14^ This study also demonstrated the presence of the components involved with the vitamin D synthesis process and the metabolites within corneal cells, and found the presence of megalin and cubilin in several types of barrier cells of the eye. The decrease in the synthesis of megalin and cubilin in rats without vitamin D receptor (VDR) is apparently associated with vitamin D secretion, which highlights the hypothesis that megalin and cubilin are involved in the metabolic pathway of vitamin D secretion into tears by cell ducts.^14^

The expression of megalin and cubilin in ocular barrier cells also indicates the importance of these receptors in vitamin D metabolism. In addition, megalin is expressed in different tissues, and the expression of cubilin is most evident in ocular barrier cells.^15^, ^16^

The vitamin D receptor was found in most tissues and cells of the human body, including corneal epithelial cells,^3^ a fact that was also observed in the study by Lin et al^4^ by demonstrating the presence of vitamin D receptor (VDR), 1‐alpha‐hydroxylase (D3‐activating enzyme), and vitamin D metabolites in the eyes. This same study showed the increased concentration of 25(OH)D3 and 24R,25(OH)D3 in tears and aqueous humor of rabbits with a vitamin D supplementation diet; thus, it can be said that a part of serum vitamin D is taken up by the ocular cells and subsequently secreted into tear fluid.

Epithelial cells of the human ocular barrier and scleral fibroblasts express receptors and enzymes necessary for vitamin D3 metabolic pathway.^3^

In a recent study, it was observed that the activity of the 25‐hydroxylase (CYP2R1) enzyme was 26 times higher in the ocular barrier cells than the 24‐hydroxylase enzyme (CYP27A1),^15^ an inactivation enzyme of 25(OH)D and of 1,25(OH)_2_D. Thus, it can be assumed that the cells of the eye can synthesize vitamin D3 directly from sun ray exposure.^3^

As the eye—like the brain—is an organ with immunological privilege, it needs efficient mechanisms to protect against infections and inflammatory responses that can cause damage. In this way, vitamin D plays an important role in the reduction of inflammation and macrophage activation in the retinal region, which leads us to suppose that it is one of the causes for the presence of a higher amount of this vitamin in tear fluid in relation to the blood.

Ocular barrier epithelial cells can convert vitamin D3 to its active form, and this synthesis has a significant value. The rate of this conversion is comparable to that of primary respiratory epithelial cells, bladder, and mammary epithelial cell lines.^3^, ^16^, ^17^ This conversion rate is much higher in tear fluid than in the collecting duct cells of the human kidney.^19^

The eye is an organ that is directly exposed to potentially harmful radiations such as ultraviolet radiation and blue light, thus the necessity of protective molecules, lutein, vitamin D, etc, to prevent or reduce possible damage to ocular tissues and cells. Since the habit of modern society of using electronic products such as smartphones, laptops, and television sets for long periods of time may increase the exposure to the radiation previously mentioned, we can therefore suppose that these acts can increase the synthesis of these protective molecules in the ocular region, leading to higher concentrations in tear fluid.

Vitamin D in the blood acts in various functions of different organs, converting into different forms and acting synergistically with other enzymes in their physiological actions. Therefore, the level this vitamin in the blood is more variable and may be lower in certain cases when compared to tear fluid, as the results of this work show. In addition, studies on chronic renal diseases have shown the reduction of megalin expression due to reduction of 25(OH)D3 level and deficient autocrine VDR activation,^20^ allowing the assumption that physiological changes may influence the serum level of vitamin D, but does not exert the same interference in the amount of vitamin D in tear fluid.

So, it is possible to determine vitamin D concentration in tears by the electrochemiluminescence method. Concentrations do not correlate with plasma vitamin D levels in the same patients. The determination of vitamin D in tear fluid by the proposed method can expand studies of ophthalmological diseases.
